# Biological functions and molecular subtypes regulated by miR-142-3p in colon cancer

**DOI:** 10.1097/MD.0000000000035422

**Published:** 2023-09-29

**Authors:** Liang Dai, Weiyan Guo, Xingwei Xuan, Baohua Wang, Haixia Chai, Guanghua Yang, Jianli Chen, Xiaodong Meng, Yinhua Wang, Jianyi Pu

**Affiliations:** a General Surgery Department, North China University of Science and Technology Affiliated Hospital, Tangshan City, China; b Intensive Care Unit, North China University of Science and Technology Affiliated Hospital, Tangshan City, China.

**Keywords:** autophagy, colon cancer, immune, metabolism, miR-142-3p

## Abstract

MicroRNA-142-3p (miR-142-3p) has been reported to be implicated in colon cancer; however, the possible regulatory mechanisms and molecular subtypes regulated by miR-142-3p have not been fully elucidated. This study aimed to investigate the biological functions and regulatory mechanism of miR-142-3p in colon cancer. The expression level of miR-142-3p in colon cancer was analyzed based on the mRNA and miRNA expression datasets of colon cancer retrieved from The Cancer Genome Atlas. Target genes of miR-142-3p were also predicted. Based on these target genes, the functions and subtypes of miR-142-3p were investigated. The metabolic and tumor-related pathways, immune microenvironment, and target gene expression between the 2 subtypes were analyzed. MiR-142-3p was upregulated in tumor tissues, and its high expression indicated a poor prognosis. A total of 39 target genes were predicted, which were significantly involved in autophagy- and metabolism-related functions and pathways. Based on these target genes, the colon cancer samples were clustered into 2 subtypes. There were 35 metabolism-related pathways that were significantly different between the 2 clusters. The immune and stromal scores in cluster 2 were higher than those in cluster 1, whereas the tumor purity of cluster 2 was significantly lower than that of cluster 1. *TP53INP2* expression in cluster 2 was higher than that in cluster 1. MiR-142-3p may promote colon cancer progression via autophagy- and metabolism-related pathways. MiR-142-3p may be served as a candidate target for the treatment of colon cancer.

**Key Points**

1. miR-142-3p was upregulated in colon cancer tumor tissues.
2. miR-142-3p may promote colon cancer progression via autophagy- and metabolism-related pathways.
3. Colon cancer samples were clustered into 2 subtypes based on the target genes of miR-142-3p.

## 1. Introduction

Colon cancer is a common malignant gastrointestinal tumor that seriously endangers human health worldwide.^[1]^ It is estimated that more than 120,000 people are diagnosed with colon cancer each year, with a mortality rate of > 33%.^[2,3]^ This disease generally begins insidiously and develops rapidly; thus, the majority of patients present with an advanced stage when they are first diagnosed.^[3]^ Despite improvements in the screening, surveillance, surgery and adjuvant chemotherapy, colon cancer remains a controversial public health challenge.^[4]^ Hence, there is an urgent requirement to identify effective biomarker for the diagnosis and treatment of colon cancer to guide its clinical management and individualized treatment.

Recently, the relative pathogenic factors of colon cancer have been widely investigated. MicroRNAs (miRNAs) are a family of RNAs with 18 to 22 nucleotides, which can lead to mRNA degradation and translation suppression by binding to target mRNAs.^[5]^ MiRNAs can regulate various molecular pathways by playing oncogenic or tumor-suppressor roles underlying the development of colon cancer.^[6]^ Recently, miR-142-3p has been found to be involved in cancer progression. The study of Gider et al has suggested that miR-142-3p is highly expressed in the peripheral blood samples of ovarian cancer patients, which may be used as the biomarker for the diagnosis, treatment and prognosis of ovarian cancer.^[7]^ Gao et al^[8]^ has revealed that miR-142-3p promotes the invasive ability of colorectal cancer cells by activating Rac family small GTPase 1. However, the possible regulatory mechanism of miR-142-3p and the molecular subtypes regulated stratified by miR-142-3p in colon cancer have not been fully investigated.

In this study, we aimed to further investigate the biological function and regulatory mechanism of miR-142-3p in colon cancer based on the colon cancer RNA expression datasets from TCGA. First, we analyzed the expression level and prognostic value of miR-142-3p in colon cancer. Next, the target genes of miR-142-3p were predicted. Based on these target genes, the functions and subtypes regulated by miR-142-3p were further analyzed, followed by subtype-regulated pathway and immune microenvironment analyses.

## 2. Data and Methods

### 2.1. Data acquisition

TCGA colon cancer expression datasets of mRNA (log2 [norm_count + 1]) and mature miRNA (log2 [RPM + 1]) based on Illumina HiSeq 2000 were downloaded from the UCSC Xene platform.^[9]^ Samples numbered –01 and –11 were selected as tumor and normal samples, respectively. Finally, 286 tumor and 41 normal samples were screened for mRNA expression. Additionally, 251 tumor and 8 normal samples were screened for miRNA expression data. Among these samples, 259 samples (8 normal and 251 tumor) were common between the mRNA and miRNA expression datasets. Moreover, the clinical and survival information for TCGA-COAD samples was downloaded.

### 2.2. Diagnostic and prognostic value of miR-142-3p in colon cancer

The miR-142-3p expression data in each sample was extracted. According to the sample grouping, the *P* value between tumor and normal samples was calculated using a t-test, and a box diagram was drawn. Moreover, based on the survival information of tumor samples (overall survival (OS) and OS time), the correlation between sample groups (miR-142-3p-high expression level ≥ cutoff value) and miR-142-3p-low (expression level < cutoff value) and survival prognosis was evaluated using the log-rank test, and a Kaplan–Meier (K–M) curve was drawn. Here, the cutoff value was obtained based on miR-142-3p expression, survival time, and survival state to detect the optimal cutoff point using R package survminer v0.4.3.

Furthermore, by combining the clinical phenotypes, including TNM stage, sex, age, tumor location, lymphatic invasion, venous invasion, and different microsatellite instability groups, the significant *P* values of miR-142-3p in different clinical phenotypes were calculated by using the t-test. Statistical significance was set at *P* < .05.

### 2.3. Differentially expressed mRNA screening

Using the classical Bayesian method provided by the limma package v3.10.3,^[10]^ differentially expressed mRNAs between tumor and normal samples were selected based on the mRNA expression (log2 [norm_count + 1]) dataset. The Benjamini and Hochberg method was used for multiple test corrections. mRNAs with |log fold change| > 1 and adjusted *P* value < 0.05 were selected as the differentially expressed mRNAs.

### 2.4. Target gene prediction for miR-142-3p

The online tool starBase v2.0^[11]^ was used for the prediction of the target gene of miR-142-3p. The threshold settings were CLIP-data ≥1 (low stringency) and degradome data ≥ 0 (with or without data). MiRNA-target relation pairs that appeared in at least one of the following prediction tools (microT, miRanda, miRmap, PITA, RNA22, PicTar, or TargetScan) were retained. The intersection of target genes and differentially expressed mRNAs was selected as the differentially expressed target genes. Pearson correlation coefficient R and significant *P* value between miR-142-3p and differentially expressed target genes were further calculated. The miR-142-3p-target pairs with R < –0.3 and adjusted *P* value < 0.05 were finally retained.

### 2.5. Functional and pathway analysis

The obtained target genes of miR-142-3p were subjected to Gene Ontology (GO) biological process (BP)^[12]^ and KEGG^[13]^ pathway analyses using the R package clusterProfiler v3.8.1,^[14]^ with *P* < .05 as the threshold. R package simplifyEnrichment v1.4.0^[15]^ was used to further process the GO BP results, because of the redundancy. This package divided the GO similarity matrix into several categories using the binary cut method, and the corresponding functions of each category were identified through annotations.

### 2.6. Identification of subtype regulated by miR-142-3p

To further reveal the possible regulatory mechanism of miR-142-3p, we clustered the samples based on the expression values of its target genes in each sample to observe whether the samples could be divided into different subgroups. Here, we applied consistent clustering to analyze the subtypes of the samples using R3.6.1 ConsensusClusterPlus v1.54.0.^[16]^ The parameter settings were maxK = 6 (maximum cluster number to evaluate); reps = 50 (number of subsamples); cluster algorithm, hc; and correlation method, Pearson. Expression heatmaps of the target genes in different subtypes were drawn.

### 2.7. Correlation analysis between subtype and clinical information

To observe the clinical characteristics and prognostic differences among the above subtypes, K–M survival curves of the different subtypes were drawn according to the prognostic information and subtype grouping of the samples. The significance *P* value was calculated using the log-rank test to determine whether there were prognostic differences. The distribution of the clinical characteristics of the different subtypes was further analyzed. For continuous variables, such as age, the *P* value was calculated using the t-test, and for categorical data, the chi-square test was applied.

### 2.8. Differential analysis of metabolic pathways and tumor-related pathways among subtypes

Based on the metabolism-related KEGG pathways in MSigDB v7.1^[17]^ and the tumor-related HALLMARK pathways as the enrichment background, we used the gene set variation analysis algorithm and R package GSVA v1.36.2^[18]^ to calculate the enrichment score of each gene set in each colon cancer sample, obtaining a scoring matrix. The limma package was used to test whether the score of each pathway was significantly different among the subtypes. Pathways with adjusted *P* value < 0.001 were considered to vary significantly between the subtypes.

### 2.9. Comparison of immune microenvironment between subtypes

Using CIBERSORT,^[19,20]^ the immune infiltration scores of 22 immune cells in each sample were calculated based on the gene expression profiles in colon cancer samples. A nonparametric test (Wilcoxon tests) was used to compare the difference of each type of immune cells between the 2 subtypes and the immune cells with a significant difference (*P* < .05) were screened. The stromal and immune scores of each tumor sample were then estimated using the ESTIMATE algorithm.^[21]^ Furthermore, the t-test was used to compare whether there were significant differences in stromal, immune, and estimated scores, and tumor purity between subtypes.

### 2.10. Expression of miR-142-3p-target gene between 2 subtypes

To further investigate the differential expression of miR-142-3p-target gene between subtypes, the t-test was used, and the genes with *P* < .05 were selected for box diagram display.

## 3. Results

### 3.1. Prognostic value of miR-142-3p in colon cancer

**Figure 1.**
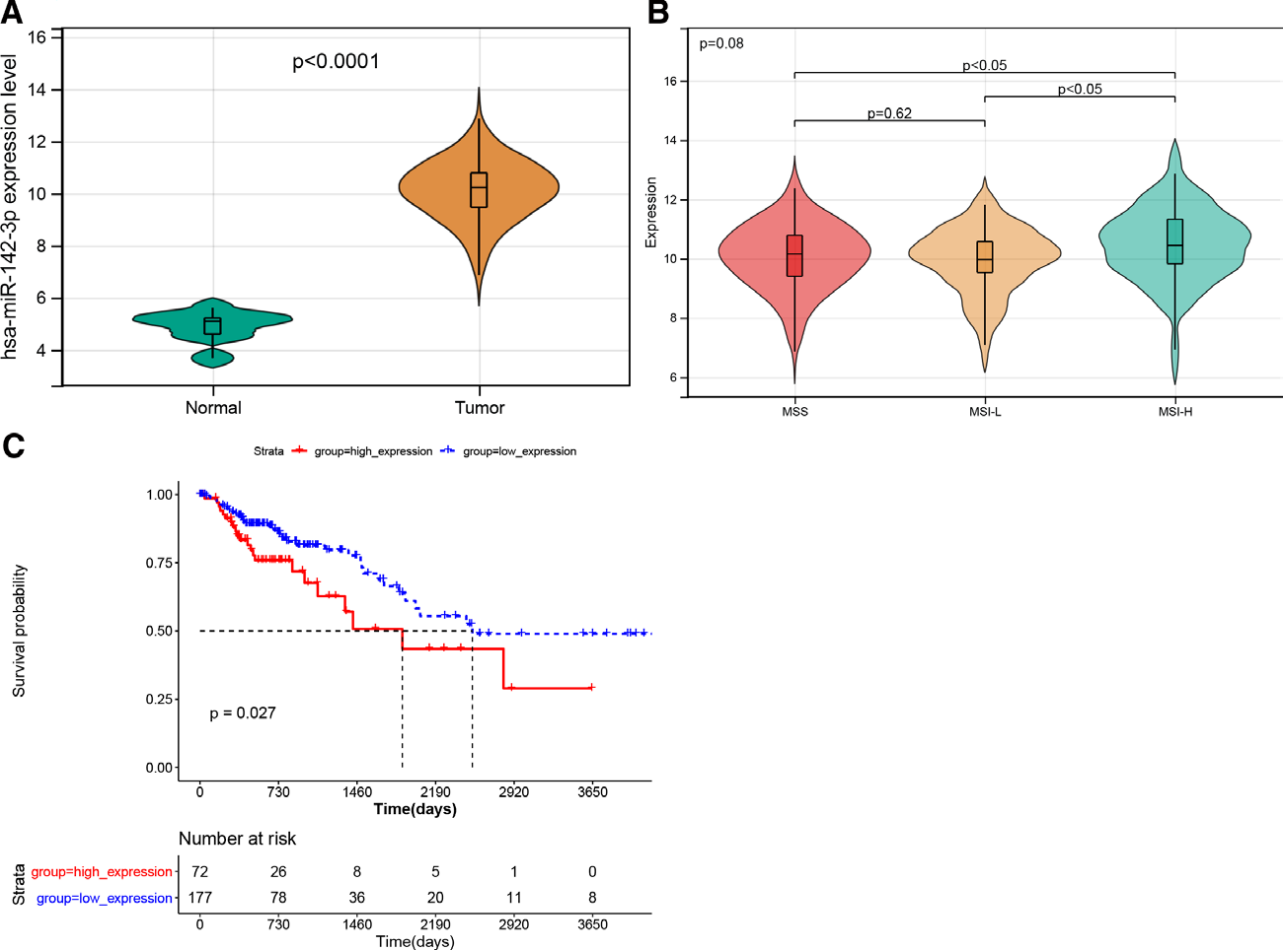
(A) Violin diagram of miR-142-3p expression in tumor and normal samples. (B) Violin diagram of miR-142-3p expression between different microsatellite instability groups. (C) Overall survival Kaplan–Meier (K–M) survival curve of miR-142-3p.

MiR-142-3p expression profiles in tumor and normal samples are shown in Figure 1A. MiR-142-3p was significantly upregulated in the tumor samples compared with normal controls. Additionally, its expression levels were differed significantly between the different microsatellite instability groups, with significantly higher expression in the high microsatellite instability group (Fig. 1B). The K–M survival curve revealed that the miR-142-3p-high expression group had a worse prognosis than that of the miR-142-3p-low expression group (Fig. 1C).

### 3.2. Differentially expressed mRNA

**Figure 2.**
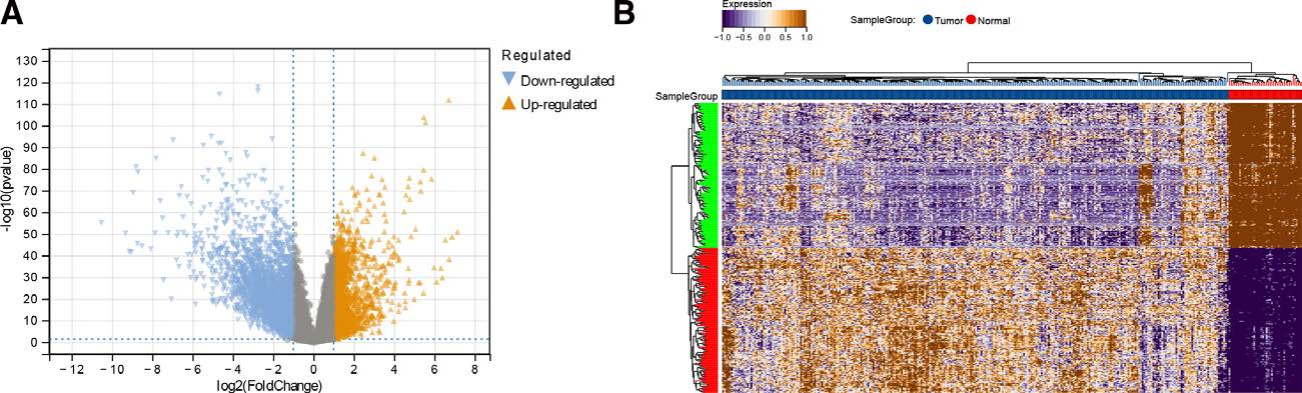
(A) Volcano plot of differentially expressed mRNAs. Blue, yellow, and gray represent downregulated genes, upregulated genes, and insignificant genes, respectively. (B) Heatmap of differentially expressed mRNAs. Blue and red at the top represent tumor and normal samples respectively, and the color change from purple to yellow in the main graph indicates an increase in expression value from small to large.

According to the screening threshold, 3238 downregulated and 1568 upregulated differentially expressed mRNAs were obtained. The differential expression patterns of mRNAs were displayed in volcano plot and heatmap (Fig. 2).

### 3.3. Target genes of miR-142-3p

**Figure 3.**
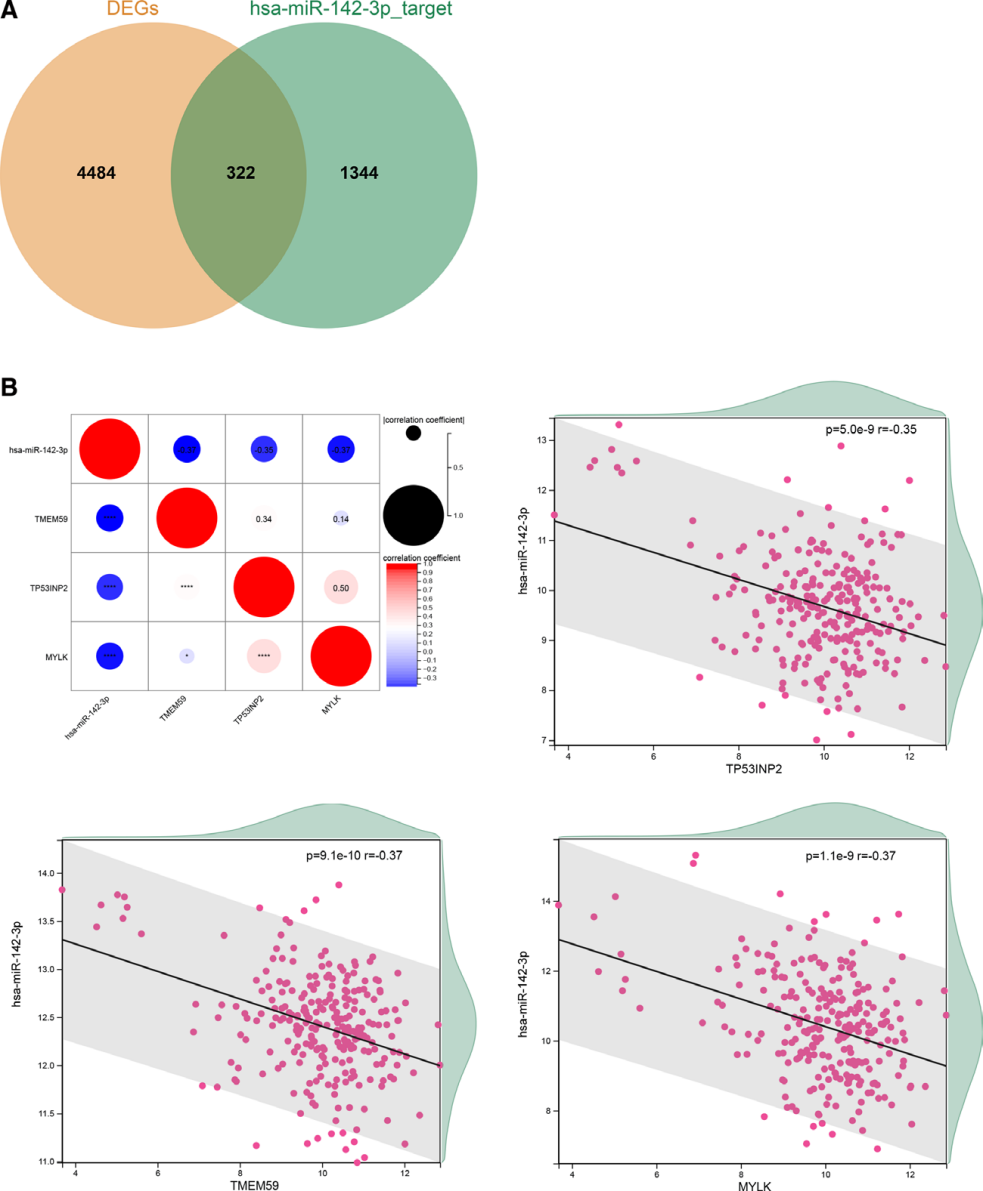
(A) Venn diagram of differentially expressed mRNAs and target genes of miR-142-3p. (B) Heatmap and scatter plot of the correlation between autophagy-related genes (*TP53INP2, TMEM59*, and *MYLK*) and miR-142-3p.

A total of 1666 target genes were predicted for miR-142-3p using starBase v2.0. After intersecting with the differentially expressed mRNAs, 322 differentially expressed target genes were identified (Fig. 3A). Next, 39 target genes were further screened according to the thresholds of R < –0.3 and adjusted *P* value < 0.05. From these targets, we selected autophagy-related genes, including tumor protein p53 inducible nuclear protein 2 (*TP53INP2*), transmembrane protein 59 (*TMEM59*), and myosin light chain kinase (*MYLK*), and drew their correlations with miR-142-3p through heatmaps and scatter plots (Fig. 3B).

### 3.4. Functional and pathway analysis for miR-142-3p

**Figure 4.**
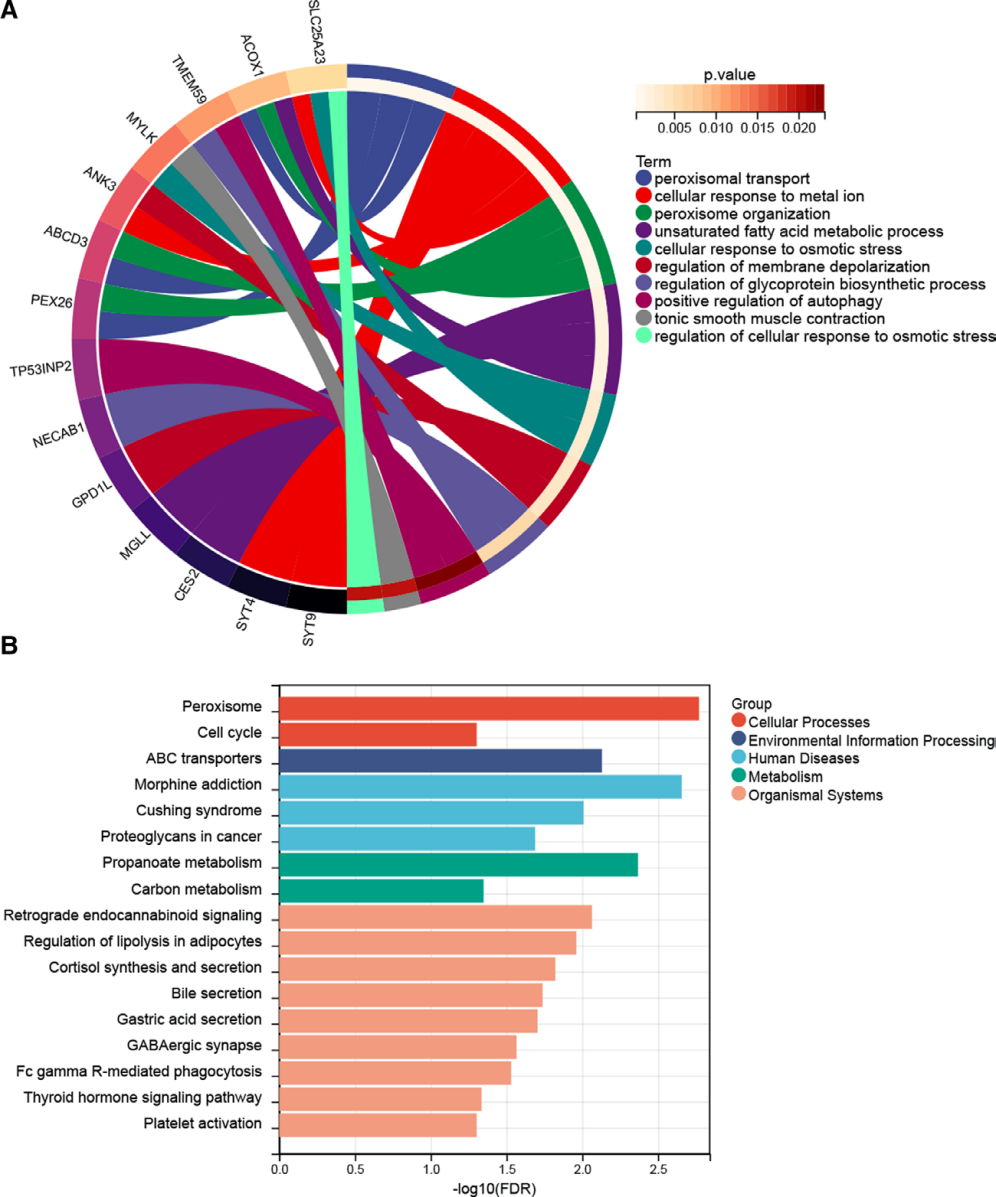
Gene Ontology biological process (A) and Kyoto Encyclopedia of Genes and Genomes pathway (B) with significant enrichment of differentially expressed target genes.

The target genes of miR-142-3p were significantly enriched in 200 GO BP terms. Further clustering analysis identified 27 functional clusters. The most significant term in each function was selected for display (Fig. 4A). Additionally, 17 significant pathways were identified. As shown in Figure 4B, these pathways are associated with autophagy and metabolism.

### 3.5. Subtype identification based on miR-142-3p-target genes

**Figure 5.**
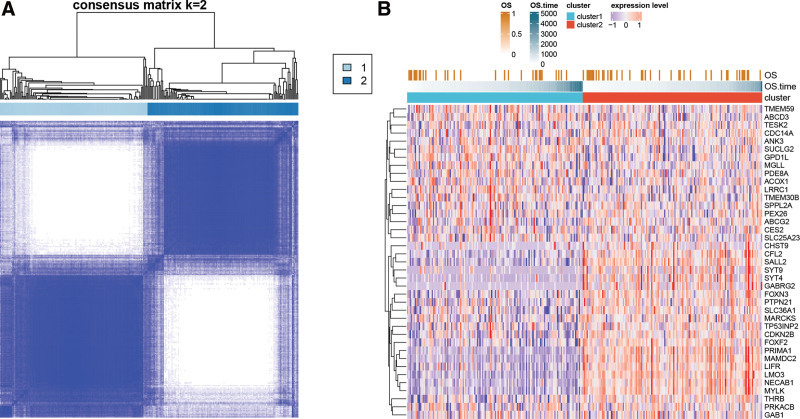
(A) Consensus clustering matrix at k = 2. (B) Heatmap of the expression of 39 target genes in different subtype samples.

Based on the expression values of the 39 target genes in each colon cancer sample, a consensus cluster analysis was conducted and identified 2 subtypes (Fig. 5A). Meanwhile, an expression heatmap of 39 genes was drawn, and it was observed that the majority of genes were highly expressed in cluster 2 (Fig. 5B).

### 3.6. Correlation analysis between subtypes and clinical information

**Figure 6.**
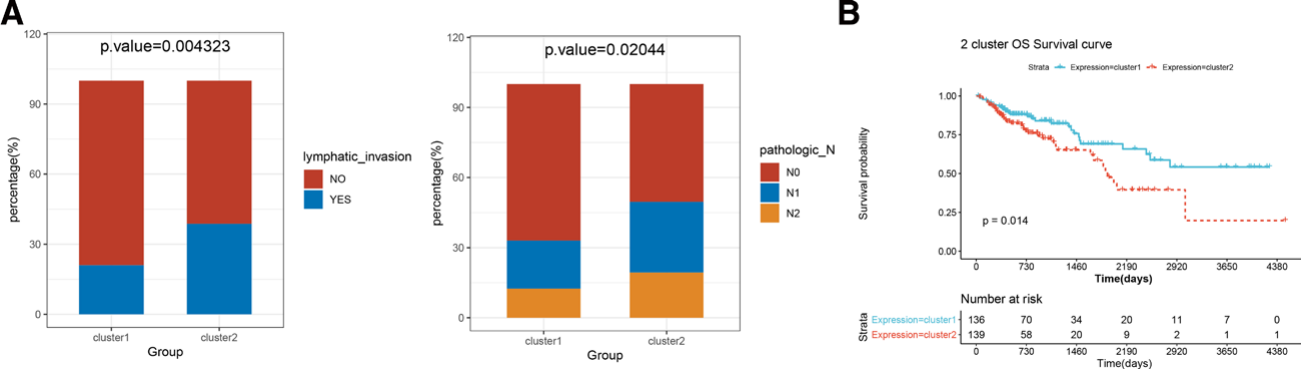
(A) Lymph node infiltration and metastasis in both subtypes. (B) Overall survival K–M survival curve of 2 subtypes.

The clinical information for the 2 subtypes is shown in Table 1. There were significant differences between the 2 subtypes in terms of lymphatic invasion and N stage (Fig. 6A). Cluster 2 contained more lymph node infiltration and N1-2 samples. Additionally, K–M survival curves for the 2 subtypes were plotted based on the prognostic information of the samples, and cluster 2 had a poor prognosis (Fig. 6B).

**Table 1:** Comparison of clinical information among subtypes.

Characteristics	Cluster 1 (N = 253)	Cluster 2 (N = 257)	Total (N = 510)	*P*
**Age**				**3.00E-04**
Mean ± SD	63.67 ± 9.91	66.89 ± 9.95	65.28 ± 10.05	
Median[min-max]	64.00 [40.00,88.00]	68.00 [33.00,87.00]	66.00 [33.00,88.00]	
**Sex**				**4.00E-04**
Female	116 (22.75%)	159 (31.18%)	275 (53.92%)	
Male	137 (26.86%)	98 (19.22%)	235 (46.08%)	
**T_stage**				**2.30E-03**
T1	64 (12.62%)	103 (20.32%)	167 (32.94%)	
T2	152 (29.98%)	124 (24.46%)	276 (54.44%)	
T3	23 (4.54%)	22 (4.34%)	45 (8.88%)	
T4	13 (2.56%)	6 (1.18%)	19 (3.75%)	
**N_stage**				0.08
N0	152 (30.52%)	175 (35.14%)	327 (65.66%)	
N1	54 (10.84%)	41 (8.23%)	95 (19.08%)	
N2	42 (8.43%)	32 (6.43%)	74 (14.86%)	
N3	2 (0.40%)	0 (0.0e + 0%)	2 (0.40%)	
**M_stage**				0.28
M0	174 (47.28%)	169 (45.92%)	343 (93.21%)	
M1	16 (4.35%)	9 (2.45%)	25 (6.79%)	
**Tumor_stage**				**0.01**
Stage I	118 (23.46%)	155 (30.82%)	273 (54.27%)	
Stage II	65 (12.92%)	55 (10.93%)	120 (23.86%)	
Stage III	51 (10.14%)	33 (6.56%)	84 (16.70%)	
Stage IV	16 (3.18%)	10 (1.99%)	26 (5.17%)	
**Prior cancer diagnosis occurrence**				0.94
No	208 (40.78%)	213 (41.76%)	421 (82.55%)	
Yes	45 (8.82%)	44 (8.63%)	89 (17.45%)	
**Location lung parenchyma**				1
Central lung	33 (17.65%)	27 (14.44%)	60 (32.09%)	
Peripheral lung	69 (36.90%)	58 (31.02%)	127 (67.91%)	
**Patient primary tumor site**				0.4
L-Lower	31 (6.26%)	45 (9.09%)	76 (15.35%)	
L-Upper	61 (12.32%)	61 (12.32%)	122 (24.65%)	
R-Lower	44 (8.89%)	50 (10.10%)	94 (18.99%)	
R-Middle	11 (2.22%)	10 (2.02%)	21 (4.24%)	
R-Upper	98 (19.80%)	84 (16.97%)	182 (36.77%)	
**Tobacco_smoking_history**				**3.00E-05**
1	24 (4.84%)	50 (10.08%)	74 (14.92%)	
2	76 (15.32%)	43 (8.67%)	119 (23.99%)	
3	54 (10.89%)	80 (16.13%)	134 (27.02%)	
4	91 (18.35%)	74 (14.92%)	165 (33.27%)	
5	3 (0.60%)	1 (0.20%)	4 (0.81%)	
**Adjuvant postoperative targeted therapy**				0.05
NO	57 (37.25%)	60 (39.22%)	117 (76.47%)	
YES	25 (16.34%)	11 (7.19%)	36 (23.53%)	
**Primary therapy outcome success type**				**1.40E-03**
CR/PR	61 (41.22%)	68 (45.95%)	129 (87.16%)	
PD/SD	17 (11.49%)	2 (1.35%)	19 (12.84%)	
**Fraction genome altered**				**8.40E-25**
Mean ± SD	0.35 ± 0.18	0.18 ± 0.16	0.27 ± 0.19	
Median[min-max]	0.35 [3.0e-4,0.78]	0.14 [0.0e + 0,0.80]	0.24 [0.0e + 0,0.80]	
**TMB (nonsynonymous**)				**1.90E-04**
Mean ± SD	9.54 ± 8.73	6.76 ± 7.30	8.00 ± 8.07	
Median[min-max]	6.47 [1.40,44.77]	4.13 [0.43,44.03]	5.33 [0.43,44.77]	

Lifelong nonsmoker (<100 cigarettes smoked in Lifetime) = 1: never smoked; current smoker (including daily smokers and nondaily smokers or occasional smokers) = 2: current smoker (including regular smokers and occasional smokers); current reformed smoker for >15 years = 3: quit smoking >15 years; current reformed smoker for ≤15 years = 4: quit smoking < 15 years; current reformed smoker, duration not specified = 5: Quit smoking, the time of quitting smoking is unknown.

### 3.7. Differential analysis of metabolic pathways and tumor-related pathways among subtypes

**Figure 7.**
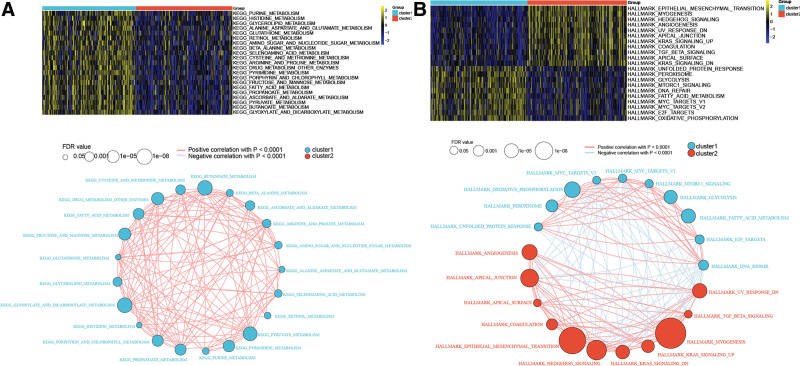
(A) Heatmaps (A) and network maps (B) of metabolism-related, and tumor-related pathways with significant differences between the 2 subtypes.

An enrichment score was obtained based on the metabolism-related KEGG pathway and tumor-related HALLMARK gene set. After comparing the 2 subtypes, a total of 35 metabolic pathways, such as pyruvate metabolism and butanoate metabolism, and 44 tumor-related pathways, such as epithelial-mesenchymal transition and myogenesis, were significantly different. Here, the metabolic pathways are presented with |t-score| >5 and cancer-related pathways with |t-score| >6 (Fig. 7A). In addition, the Spearman correlation coefficient between these pathways was calculated and a network was drawn. As shown in Figure 7B, metabolism-related pathways have higher overall enrichment scores in cluster 1.

### 3.8. Comparison of immune microenvironment between subtypes

**Figure 8.**
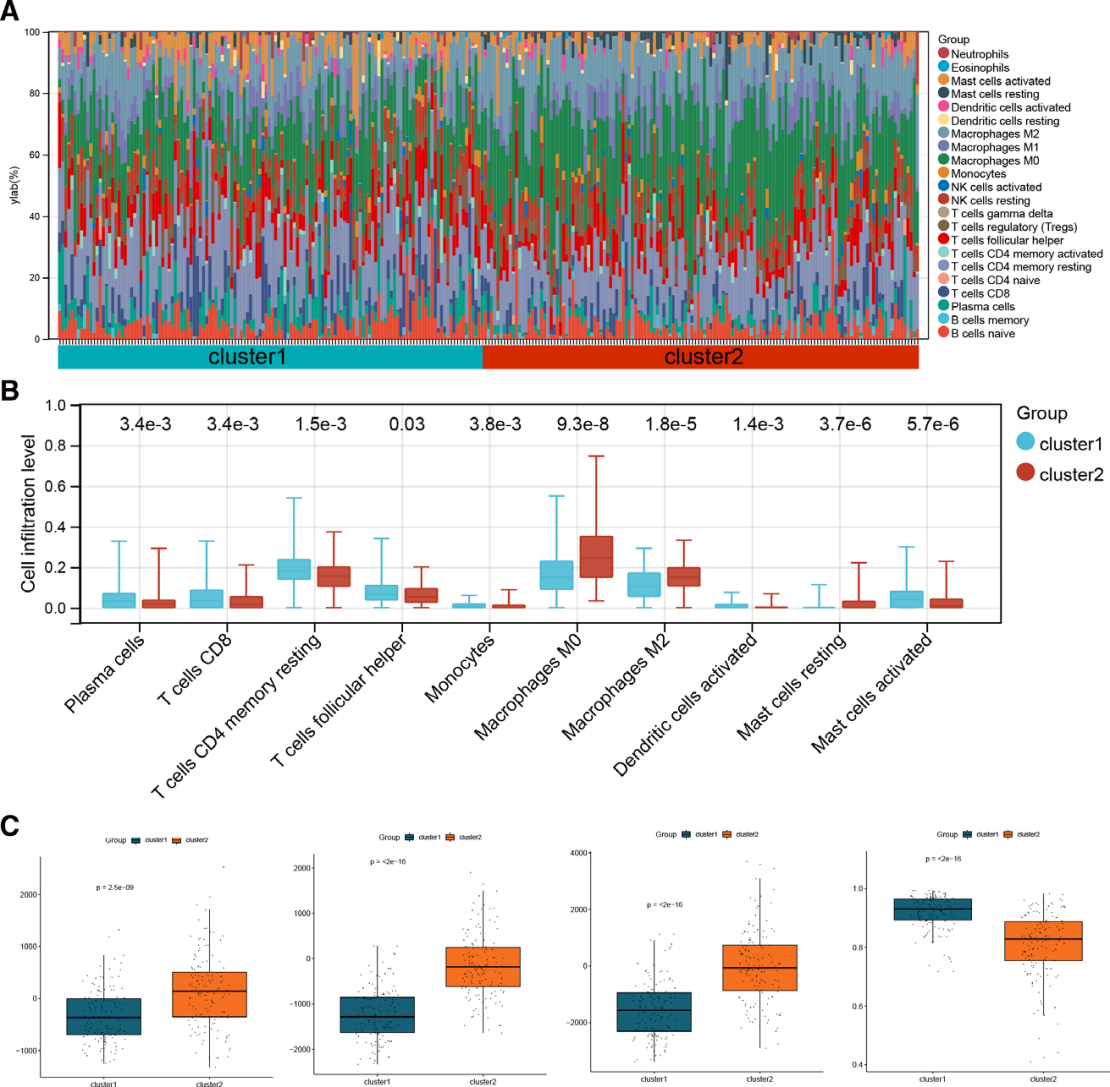
(A) Histogram of horizontal accumulation of the infiltration level of 22 types of immune cells. (B) Box plot of cell infiltration levels with significant differences between the 2 subtypes. (C) Box plots of the difference of immune score, stromal score, microenvironment score, and tumor purity between the 2 subtypes.

The relative infiltration levels of 22 immune cells are shown in Figure 8A. Additionally, immune cells with significantly differential infiltration levels between the 2 subtypes were extracted. Macrophages M0 and M2 had high infiltration levels in cluster 2 (Fig. 8B). The immune and stromal scores in cluster 2 were higher than those in cluster 1, whereas the tumor purity in cluster 2 was lower than that in cluster 1 (Fig. 8C).

### 3.9. Expression of miR-142-3p-target genes between the 2 subtypes

**Figure 9.**
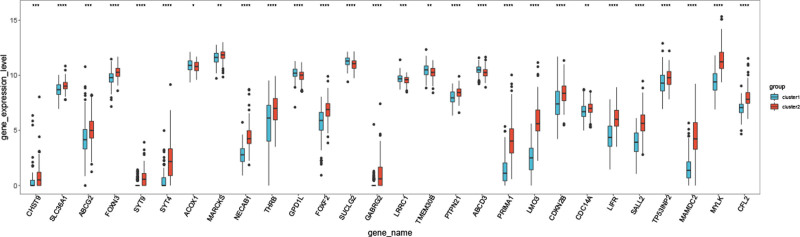
Box diagram of miR-142-3p-target genes with significant differences between the 2 subtypes.

The expression levels of 28 genes, including *TP53INP2* and *MYLK*, were significantly different between the 2 subtypes (Fig. 9).

## 4. Discussion

In the present study, based on TCGA dataset, miR-142-3p was upregulated in colon cancer tumor tissues. A total of 39 target genes were predicted, including 3 autophagy-related genes (*TP53INP2, TMEM59*, and *MYLK*). These target genes were significantly involved in autophagy- and metabolism-related functions and pathways. Moreover, based on these target genes, the tumor samples were clustered into 2 subtypes, and cluster 2 had a worse prognosis than that of cluster 1. In addition, the immune and stromal scores in cluster 2 were higher than those in cluster 1, whereas the tumor purity of cluster 2 was lower than that of cluster 1.

MiRNAs are key posttranscriptional regulators and are involved in carcinogenesis.^[22]^ MiR-142-3p, located on chromosome 17q22, participates in many physiological and pathological processes, like hematopoietic stem cell differentiation and tumorigenesis.^[23,24]^ miR-142-3p is implicated in the carcinogenesis of several human cancers, including colon cancer.^[23,25–27]^ Consistent with the study by Zheng et al,^[27]^ we found that miR-142-3p was upregulated in tumor tissues of colon cancer, and its high expression was associated with a poor prognosis.

Autophagy is an essential process that degrades and recycles cellular components, and its dysregulation is associated with various human diseases.^[28–30]^ MiR-142-3p can regulate tumor cell autophagy during the progression of colon cancer by regulating *TP53INP2*.^[27]^ In the present study, the target genes (*TMEM59* and *TP53INP2*) of miR-142-3p were significantly enriched in functions related to positive regulation of autophagy. TMEM59 can induce unconventional autophagy in response to bacterial infection.^[31]^ However, its role in cancer remains unclear. TP53INP2 is a dual regulator of transcription and enhances autophagy.^[32]^ It interacts with the transmembrane protein VMP1, contributing to the formation of autophagosome.^[33]^ Importantly, *TP53INP2* has been reported to be implicated in several human cancers, including colon cancer.^[27,34,35]^ Taken together, our study suggested that miR-142-3p may promote colon cancer progression by regulating autophagy.

To further elucidate the possible regulatory mechanism of miR-142-3p, we clustered the samples into 2 subtypes. The results showed that cluster 2 had a worse prognosis than that of cluster 1. Interestingly, we found that cluster 2 contained more samples with lymph node infiltration and N1-2, which may be responsible for its poor prognosis.

Metabolism refers to chemical reactions that provide energy for life-sustaining activities and assimilation of new substances.^[36]^ Metabolism alteration is a hallmark of cancers, contributing to the initiation, development, and maintenance of malignant transformation.^[37]^ In the present study, 35 metabolism-related pathways, such as pyruvate and butanoate metabolism, were significantly different between the 2 subtypes. Therefore, abnormal pyruvate metabolism plays a prominent role in human cancers.^[38]^ Additionally, the results showed that metabolism-related pathways had a higher overall enrichment score in cluster 1. Therefore, the identification of these differential metabolic pathways may offer a new avenue for therapeutic intervention in patients in cluster 1.

The tumor immune microenvironment is a complex system.^[39]^ Cancer immunotherapy will be the main treatment method for patients with cancer in the future, thus understanding the heterogeneity of the tumor immune microenvironment is crucial. The present study compared the immune microenvironments of the 2 colon cancer subtypes. Macrophages M0 and M2 showed high levels of infiltration in cluster 2. The interaction between macrophages and tumor cells is complex. Under certain circumstances, activated macrophages exhibit antitumor activity. However, an increased density of macrophages in the tumor stroma is associated with poor prognosis in certain solid tumors.^[40]^ Tumor-associated macrophages coordinate various factors in the tumor microenvironment to promote tumor progression.^[41]^ In addition to immune cell infiltration, immune score can also reflect the immune microenvironment.^[42,43]^ In the present study, there were significant differences between the 2 subtypes. Thus, we speculate that miR-142-3p may serve as a target in colon cancer immunotherapy.

In conclusion, miR-142-3p was upregulated in colon cancer tumor tissues, indicating poor prognosis. MiR-142-3p may promote the progression of colon cancer via autophagy- and metabolism-related pathways. Moreover, the colon cancer samples could be divided into 2 subtypes, and there were significant differences between the 2 subtypes with respect to prognosis, metabolism-related pathways, immune microenvironment, and target gene expression. Our results further suggest that miR-142-3p may be served as a promising target for colon cancer treatment.

## Acknowledgments

We acknowledge TCGA database for providing their platforms and contributors for uploading their meaningful datasets.

## Author contributions

**Conceptualization:** Liang Dai, Jianyi Pu.

**Data curation:** Weiyan Guo.

**Formal analysis:** Liang Dai, Weiyan Guo, Xingwei Xuan, Jianli Chen, Yinhua Wang.

**Investigation:** Xingwei Xuan.

**Methodology:** Weiyan Guo, Guanghua Yang.

**Project administration:** Jianyi Pu.

**Resources:** Xingwei Xuan, Baohua Wang, Guanghua Yang, Jianli Chen.

**Software:** Baohua Wang, Haixia Chai, Xiaodong Meng.

**Supervision:** Jianyi Pu.

**Visualization:** Haixia Chai.

**Writing—original draft:** Liang Dai, Yinhua Wang.

**Writing—review & editing:** Baohua Wang, Xiaodong Meng.
